# Effects of Soy Isoflavones and Green Tea Extract on Simvastatin Pharmacokinetics and Influence of the SLCO1B1 521T > C Polymorphism

**DOI:** 10.3389/fnut.2022.868126

**Published:** 2022-05-19

**Authors:** Weiwei Zeng, Miao Hu, Hon Kit Lee, Elaine Wat, Clara Bik San Lau, Chung Shun Ho, Chun Kwok Wong, Brian Tomlinson

**Affiliations:** ^1^The Second People’s Hospital of Longgang District, Shenzhen, China; ^2^Shenzhen Baoan Women’s and Children’s Hospital, Jinan University, Shenzhen, China; ^3^Department of Medicine and Therapeutics, Prince of Wales Hospital, The Chinese University of Hong Kong, Hong Kong SAR, China; ^4^Department of Chemical Pathology, The Chinese University of Hong Kong, Hong Kong SAR, China; ^5^Department of Clinical Pathology, Tuen Mun Hospital, Hong Kong, Hong Kong SAR, China; ^6^State Key Laboratory of Research on Bioactivities and Clinical Applications of Medicinal Plants, Institute of Chinese Medicine, The Chinese University of Hong Kong, Hong Kong SAR, China; ^7^Faculty of Medicine, Macau University of Science and Technology, Taipa, Macao SAR, China

**Keywords:** SLCO1B1, drug interaction, EGCG, green tea, simvastatin, soy isoflavones

## Abstract

**Objectives:**

Green tea and soy products are extensively consumed by many people and they may influence the activity of drug metabolizing enzymes and drug transporters to result in drug interactions. This study was performed to evaluate the effect of green tea and soy isoflavone extracts on the pharmacokinetics of simvastatin in healthy subjects and to clarify the role of polymorphisms in the SLCO1B1 drug transporter in this effect.

**Methods:**

This was an open-label, three-phase randomized crossover pharmacokinetic study. A single dose of simvastatin 20 mg was taken on three occasions (without herbs, with green tea, and with soy isoflavones) by healthy male Chinese subjects. The green tea and soy isoflavone extracts were given at a dose containing EGCG 800 mg once daily or soy isoflavones about 80 mg once daily for 14 days before simvastatin dosing with at least 4-weeks washout period between phases.

**Results:**

All the 18 subjects completed the study. Intake of soy isoflavones was associated with reduced systemic exposure to simvastatin acid [geometric mean (% coefficient of variation) AUC_0–24h_ from 16.1 (44.2) h⋅μg/L to 12.1 (54.6) h⋅μg/L, *P* < 0.05) but not the lactone. Further analysis showed that the interaction between simvastatin and the soy isoflavones only resulted in a significant reduction of AUC in subjects with the SLCO1B1 521TT genotype and not in those with the 521C variant allele. There was no overall effect of the green tea extract on simvastatin pharmacokinetics but the group with the SLCO1B1 521TT genotype showed reduced AUC values for simvastatin acid.

**Conclusion:**

This study showed repeated administration of soy isoflavones reduced the systemic bioavailability of simvastatin in healthy volunteers that was dependent on the SLCO1B1 genotype which suggested that soy isoflavones-simvastatin interaction is impacted by genotype-related function of this liver uptake transporter.

## Introduction

Cardiovascular diseases (CVDs) are a significant health burden with an increasing prevalence and remain the leading causes of morbidity and mortality worldwide (1). Use of herbal medicines and foods and beverages thought to have beneficial effects in CVD is very common among patients with this condition (2). There may be interactions between herbal medicines or foods with the drugs taken for CVD which may result in toxicity or altered efficacy. The composition of most herbal medicines is complex, with each herb containing a variety of chemical components and each of these components may lead to herb-drug interactions by affecting the activity of drug metabolizing enzymes or drug transporters (3).

Simvastatin is one of the 3-hydroxy-3-methylglutaryl coenzyme A (HMG-CoA) reductase inhibitors or statins, which has been used extensively worldwide to reduce low-density lipoprotein cholesterol (LDL-C) and the risk for CVD events. It is administered in the inactive lactone form and is rapidly hydrolyzed to the active open acid form, simvastatin acid (4). This is converted back to simvastatin lactone through a glucuronidation and lactonization pathway (5). Simvastatin acid and lactone are extensively metabolized by cytochrome P450 (CYP) enzymes, mainly CYP3A4 and CYP3A5 with a minor contribution from CYP2C8 (6). Simvastatin acid is a substrate for the liver uptake transporter organic anion–transporting polypeptide 1B1 (OATP1B1) encoded by SLCO1B1 and the adenosine triphosphate (ATP)-binding cassette (ABC) efflux transporters, ABCG2 and ABCB1 (7). The ABCG2 c.421C > A polymorphism contributed toward differences in exposure to simvastatin acid between Caucasian and Asian subjects (8).

The SLCO1B1 c.521T > C single nucleotide polymorphism (SNP) can result in marked inter-individual differences in pharmacokinetics of simvastatin acid (9) and was the only functional genetic variant associated with simvastatin-induced myopathy in a genome wide association study (GWAS) (10) and this variant also influenced the lipid response to simvastatin in a meta-analysis of GWAS (11). A GWAS of the LDL-C response to simvastatin in the Heart Protection Study also identified an ABCC2 variant having a significant effect (12). Drugs and chemicals from herbs and food materials which interact with any of these pathways could therefore influence the systemic exposure and efficacy and safety of simvastatin. A case was reported in which green tea was thought to interact with simvastatin to cause muscle pain (13).

Soybeans contain large amounts of isoflavones, including phytoestrogens, and there is evidence that some isoflavones may modify CYP enzyme expression and activity (14). The average daily intake of soy foods in Asian adults provides an average of 15-45 mg isoflavones/day (15) Rats fed diets containing soy protein isolate showed increased activity and expression of CYP3A1 (16) related to greater binding of the pregnane X receptor (PXR) to a response element on the CYP3A1 promoter (17). Isoflavones also activated human PXR increasing CYP3A4 expression (18). Soy components can activate other nuclear receptors including peroxisome-proliferator activated receptors (PPAR) α and PPARγ and liver X receptor (LXR) resulting in increased expression of CYP3As (19–21). These nuclear receptors can also modulate the expression of drug transporters.

On the other hand, flavonoids can inhibit multiple ABC efflux transporters, including ABCB1, ABCC2 and ABCG2 (22). Many flavonoids can inhibit OATP1B1 in a concentration-dependent manner but rutin had a stimulatory effect (23). In a recent study which investigated the effect of 25 common flavonoids on OATP1B1-mediated uptake of the fluorescent substrate 2′,7′-dichlorofluorescein (DCF) in Chinese hamster ovary (CHO) cells stably expressing human OATP1B1, most of the flavonoids tested had a concentration-dependent inhibitory effect on OATP1B1-mediated DCF uptake, but a low concentration of epicatechin gallate (ECG) showed a stimulating effect of about 160% (24).

This study was conducted to examine the effect of green tea extract and soy isoflavones on the pharmacokinetics of simvastatin in healthy subjects and whether any interactions were influenced by the polymorphisms in SLCO1B1.

## Materials and Methods

### Subjects

Eighteen healthy Chinese male subjects who gave written informed consents were recruited for the study. Participants were selected from a group of healthy subjects who had previously been genotyped for the SLCO1B1 521T > C (rs4149056) polymorphism so there would be a reasonable number of subjects in each of the 3 genotype groups. The study was performed in accordance with the ethical standards laid down in the Declaration of Helsinki and approved by the Joint Chinese University of Hong Kong-New Territories East Cluster Clinical Research Ethics Committee with reference number CRE-2010.524-T.

Subjects were required to abstain from taking any prescription or non-prescription medications from 2 weeks prior to and during the study. Smoking, alcohol, grapefruit juice, caffeine, soybean milk, tea, dietary supplements, and herbal products were forbidden from 2 weeks before and throughout the entire study. Subjects fasted for 10 h before and 4 h after administration of simvastatin during the blood sampling sessions. Standardized meals were provided to consume at 4 h and 10 h after drug administration. The meals were provided by the hospital catering service and were the same for all subjects and the same for the 3 phases of the study. Water intake was not allowed from 1 h pre-dose to 1 h post-dose except for the water provided for drug administration. Subjects were asked to report any adverse effects during the pharmacokinetic sampling and at other visits to the study center.

### Simvastatin-Herb Pharmacokinetic Interaction

The study was an open-label, three-phase randomized crossover design. Simvastatin 20 mg (Zocor^®^, MSD) was given 3 times: 1. simvastatin only; 2. with green tea extract; 3. with soy isoflavones extract. There was a washout period of at least 4 weeks between phases. The extracts of green tea or soy isoflavones were taken as one sachet once daily in the morning before breakfast for 14 days. The herbal extracts were provided as a powder which was taken in 150 ml water at room temperature. Fourteen blood samples were taken at 0, 0.25, 0.5, 1, 1.5, 2, 2.5, 3, 4, 6, 8, 10, 12, and 24 h after the dose to evaluate the pharmacokinetic profile on the simvastatin dosing days. A food diary was used to monitor the food compliance during the study and subjects were asked to record all their daily food intake including the main meals, snacks and beverages. Subjects were requested to follow the requirements on diet carefully.

### Herbal Products

The extracts of green tea and soy isoflavones were manufactured by the Hong Kong Institute of Biotechnology (HKIB) in accordance with Good Manufacturing Practice (GMP). Standard heavy metals, microbial and pesticide testing was performed to ensure the products fulfilled the safety requirements set out by the Department of Health in Hong Kong. Each sachet of green tea extract or soy isoflavones was claimed to contain 800 mg standardized polyphenol (mainly EGCG) or 120 mg total isoflavones, respectively.

### Establishment of Chemical Profiles of Herbal Products

Ultra-Performance Liquid Chromatography (UPLC) methods were used to verify the contents of the green tea extracts and soy extracts in the laboratory of the Institute of Chinese Medicine, the Chinese University of Hong Kong as described previously (25). The green tea extract was compared with a standard mixture containing 7 chemical markers: gallic acid (GA), epigallocatechin (EGC), catechin (C), epigallocatechin gallate (EGCG), caffeine (CAF), epicatechin (EC) and epicatechin gallate (ECG) in methanol. Soy isoflavone extract was compared with a standard mixture containing 7 chemical markers: glycitin, daidzin, genistin, daidzein, glycitein, genistein and acetylgenistin in methanol.

### Quantification of Plasma Concentrations of Simvastatin and Simvastatin Acid

Plasma concentrations of simvastatin and its major active metabolite simvastatin acid were determined by Liquid Chromatography-Tandem Mass Spectrometry (LC-MS/MS) employing online sample pre-treatment.

The concentrations of simvastatin acid and lactone in plasma were simultaneously quantified by a method validated according to the U.S. Food and Drug Administration (USFDA) guidance on Bioanalytical Method Validation (26) employing LC-MS/MS using the corresponding isotopically labeled compounds as internal standards. The plasma samples were prepared using liquid–liquid extraction with diethyl ether. Chromatographic separation was accomplished on an XBridge C18 (3.5 μm 2.1 × 30 mm Column; Waters, MA, United States). qqThe mobile phase consisted of a gradient mixture of 0.015 mmol/L ammonium acetate in water (mobile phase A) and methanol (mobile phase B) at a flow rate of 0.4 mL/min. The gradient started at 50% mobile phase B for 0.5 min with a subsequent fast gradient to 98% mobile phase B in 1 min and maintained for another 0.5 min. The gradient was then returned to the initial mobile phase concentration in a chromatographic run of 3 min. Simvastatin acid was detected in a negative ionization mode with a quantification transition of m/z 435.4–115.0 and a qualification transition of m/z 435.4–319.2 while the lactone was quantified in a positive ionization mode with a transition of m/z 441.3–310.2 and monitored by a qualification transition of m/z 441.3–310.2. The lower limits of quantification of simvastatin acid and lactone were 0.1 μg/L by using 300 μL plasma. The linear ranges of the method were from 0.1 to 20.0 μg/L for both simvastatin acid and lactone. The coefficients of variation were lower than 10 and 9% for simvastatin acid and lactone, respectively.

### Effects on Plasma Lipid Profile and Blood Pressure With Green Tea Extract and Soy Isoflavones

Fasting lipid profiles and blood pressure were monitored at baseline and after 14 days of green tea extract and soy isoflavones, respectively. Plasma lipid profile including total cholesterol, triglycerides, and high-density lipoprotein cholesterol (HDL-C) was measured on a Roche Modular Analytics system (Roche Diagnostics GmbH, Mannheim, Germany) using standard reagent kits supplied by the manufacturer of the analyzer and LDL-C level was estimated by using the Friedewald formula (27) or directly measured when the triglyceride level was over 4.5 mmol/L. After 5 min of resting seated, clinic blood pressure and heart rate was measured four times at 2-min intervals in the dominant arm with an automatic device (Omron HEM 7080IT, Omron Healthcare). The average of the last three measurements was used in the statistical analyses.

### Genotyping

High Pure PCR Template Preparation Kits (Roche Applied Science) were used to extracted DNA from the blood samples. TaqMan Drug Metabolism Genotyping Assays from Applied Biosystems (Foster City, CA, United States) was used to genotype each subject for the SLCO1B1 388A > G (rs2306283) and 521T > C (rs4149056) polymorphisms.

### Pharmacokinetic Analysis

The pharmacokinetic parameters of simvastatin and its active metabolite simvastatin acid were calculated using non-compartmental methods with the aid of the computer program WinNolin (version 2.1, Pharsight Corporation). The maximum plasma concentration (C_max_) and time to C_max_ (t_max_) were obtained directly from the observed concentration-time data. The terminal elimination rate constant (λ_Z_) was determined by linear regression of the terminal portion of the concentration-time curve and the elimination half-life (t_1/2_) was calculated as 0.693/λ_Z_.

Systemic exposure to simvastatin lactone and simvastatin acid was evaluated by calculating the AUC using the linear trapezoidal rule and AUC_0–∞_ was calculated as AUC_0–∞_ = AUC_0–t_ + C_t_/K_el_ where C_t_ is the last quantifiable concentration. The oral clearance (CL/F) was calculated as Dose/AUC_0–∞_.

### Statistical Analysis

The pharmacokinetic parameters of simvastatin with and without herb consumptions calculated by repeated measures ANOVA and the Friedman rank test was used to compare t_max_ values. The geometric mean ratios and 90% confidence intervals (CI) were calculated from the log-transformed values of C_max_ and AUC compared between with and without the herbal extracts. ANOVA analysis was used to compare the pharmacokinetic parameters and interactions among genotype groups if the data was normally distributed, otherwise the Kruskal-Wallis test was used for skewed data. P < 0.05 was considered statistically significant.

### Sample Size

Previous studies have shown that significant effects of polymorphisms in drug transporters can be seen for single-dose complete pharmacokinetic studies in small groups of *n* = 6 (28). A herb-drug interaction between baicalin and rosuvastatin was shown to be related to different SLCO1B1 haplotype groups in 18 healthy Chinese subjects, with 6 subjects in each haplotype group. A similar sample size of 18 subjects was used in the current study to explore the potential herb-drug interactions and their relationship with the SLCO1B1 521T > C (rs4149056) polymorphism.

## Results

### Establishment of Chemical Profiles of Herbal Products

The green tea extract contained mainly EGCG and the other 6 chemical markers were present in small amounts. Each sachet continued the amounts of EGCG, ECG, EC, EGC, GA, CAF, and C of 804.6, 45.5, 5.9, 3.7, 1.02, 1.08, and 0.96 mg, respectively.

The soy isoflavone product contained the 7 chemical markers, namely glycitin, daidzin, genistin, daidzein, glycitein, genistein and acetylgenistin in amounts of 58.64, 8.72, 6.48, 2.20, 4.23, 0.42, and 0.90 mg per sachet, respectively. These seven isoflavones were calculated to contribute a total of 81.6 mg per sachet. Some other small unidentified peaks were seen on the chromatogram and these may represent other components of the extract that contribute to the total isoflavones.

### Effect of Green Tea Extract and Soy Isoflavones on the Pharmacokinetics of Simvastatin and Simvastatin Acid

In the 18 healthy Chinese male volunteers (mean (± SD) age: 26.6 ± 6.0 years; body weight: 61.7 ± 6.3 kg; body mass index: 21.1 ± 1.7 kg/m^2^) (Table 1), intake of green tea extract 800 mg daily for 14 days had no significant effect on the average pharmacokinetic parameters for simvastatin or simvastatin acid (Table 2). However, intake of soy isoflavones significantly reduced the systemic exposure to simvastatin acid with significant reductions in the AUC values but not in C_max_ (Table 2). There was no significant effect on any of the pharmacokinetic parameters for simvastatin lactone with the green tea or soy extracts.

**TABLE 1:** Demographics of the 18 study subjects in the simvastatin-herb interaction study.

Demographics	All subjects (*n* = 18)	SLCO1B1 521TT (*n* = 6)			SLCO1B1 521TC (*n* = 8)			SLCO1B1 521CC (*n* = 4)		
		388AA	388AG	388GG	388AA	388AG	388GG	388AA	388AG	388GG
		(*n* = 0)	(*n* = 3)	(*n* = 3)	(*n* = 1)	(*n* = 4)	(*n* = 3)	(*n* = 0)	(*n* = 0)	(*n* = 4)
Age, years	26.6 ± 6.0		26.7 ± 6.4	26.3 ± 1.5	28	22.5 ± 1.7	36.7 ± 5.1			25.3 ± 5.1
BW, kg	61.7 ± 6.3		62.5 ± 6.2	60.8 ± 2.4	64.2	58.0 ± 8.6	66.1 ± 8.2			55.2 ± 5.6*
BMI, kg/m^2^	21.1 ± 1.7		20.8 ± 1.9	20.5 ± 1.1	22.9	20.5 ± 2.4	22.2 ± 2.4			19.8 ± 1.7*

*P < 0.05 vs. SLCO1B1 521TT; BW, body weight.

**TABLE 2:** Effect of green tea extract and soy isoflavones on the pharmacokinetic parametes of simvastatin lactone and simvastatin acid in 18 healthy subjects.

Variable	Simvastatin	Simvastatin and Green tea	Simvastatinand Soy isoflavones	Overall *P* values
**Simvastatin lactone**				
C_max_, μg/L	3.35 (54.9)	3.68 (48.1)	3.64 (32.3)	0.814
GMR (90%CI)		0.95 (0.95–1.72)	0.97 (0.92–1.74)	
AUC_0–24 h_	14.4 (49.7)	16.14 (40.5)	14.39 (33.7)	0.319
GMR (90%CI)		1.06 (1.00–1.52)	1.04 (0.90–1.29)	
AUC_0–∞_	14.7 (48.6)	17.17 (39.8)	15.2 (35.9)	0.211
T_1/2_, h	4.47 (27.4)	4.96 (34.0)	4.51 (33.6)	0.772
T_max_, h	1.0 (0.875, 2.5)	1.5 (1.0, 2.25)	2.0 (1.375, 2.25)	0.138
**Simvastatin acid**				
C_max_, μg/L	2.21 (50.4)	1.81 (50.0)	1.78 (53.6)	0.427
GMR (90%CI)	–	0.93 (0.67–1.40)	0.82 (0.73–1.26)	
AUC_0–24h_	16.09 (44.2)	14.78 (48.1)	12.09 (54.6)*	0.015
GMR (90%CI)	–	0.85 (0.77–1.27)	0.73 (0.68–0.95)	
AUC_0–∞_	17.76 (45.3)	16.53 (45.8)	12.82 (54.6)*	0.010
T_1/2_, h	3.94 (31.7)	5.17 (38.7)	3.89 (28.3)	0.262
T_max_, h	4.0 (3.0, 4.0)	4.0 (2.88, 4.0)	4.0 (3.0, 4.0)	0.976

*Data are geometric mean (% CV) except for T_max_ for which median (IQR) are given.*

*P < 0.05 vs. baseline. AUC values in h⋅μg/L.

*GMR, geometric mean ratio compared to simvastatin alone value.*

**FIGURE 1:**
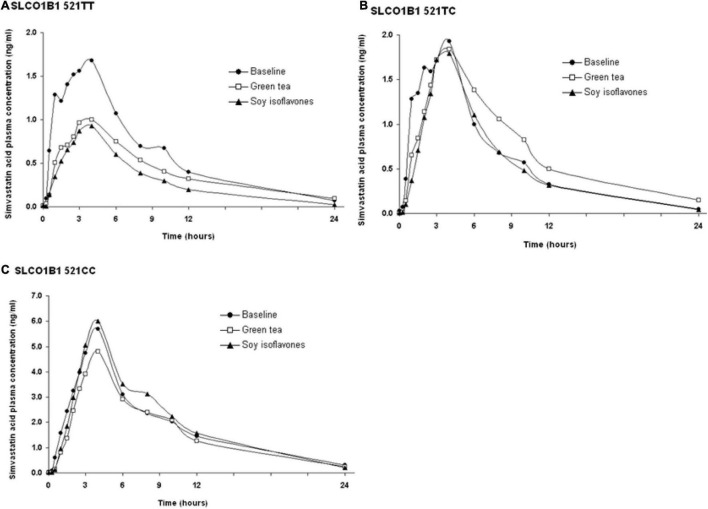
Effects of the SLCO1B1 521 T > C polymorphism on the pharmacokinetic interactions between simvastatin acid before and after the green tea extract and soy isoflavones. Data are arithmetic mean.

**FIGURE 2:**
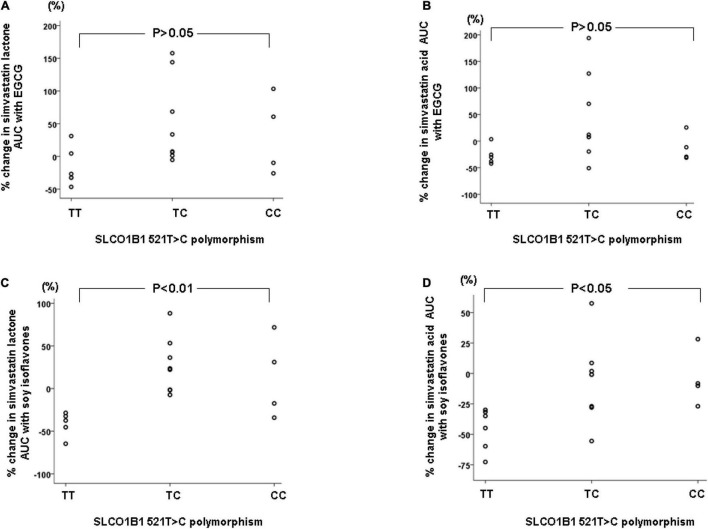
Percentage changes in AUC_0–24h_ of simvastatin and simvastatin acid with green tea extract **(A,B)** or soy isoflavones **(C,D)** according to the SLCO1B1 521T > C genotypes.

The SLCO1B1 521T > C polymorphism was significantly associated with the pharmacokinetics of simvastatin acid, but not the lactone. Compared to the other genotype groups, subjects with the 521CC genotype (*n* = 4) had increased systemic exposure to simvastatin acid, but not simvastatin lactone, at baseline and after intake of green tea and soy isoflavones and the difference remained statistically significant after adjustment for body weight (Tables 3, 4). Further analysis showed that the interaction between simvastatin acid and the soy isoflavones only occurred in subjects with the 521TT genotype but not in those with the TC or CC genotypes (Table 4 and Figure 1). The percentage reduction in AUC_0–24h_ of simvastatin lactone and simvastatin acid with soy isoflavones in subjects with 521TT genotype were significantly greater than those with CC genotypes (Figure 2).

**TABLE 3:** Effects of the SLCO1B1 521 T > C polymorphism on the pharmacokinetics of simvastatin lactone at baseline and after intake of the green tea extract and soy isoflavones.

Variable	521TT (*n* = 6)	521TC (*n* = 8)	521CC (*n* = 4)	*P* values
**Baseline**				
C_max_, μg/L	4.21 (63.5)	3.4 (55.0)	2.3 (47.8)	>0.05
AUC_0–24h_, h⋅μg/L	24.1 (50.1)	9.5 (35.9)	15.0 (42.5)	>0.05
AUC_0–∞_, h⋅μg/L	24.4 (55.8)	10.3 (35.2)	16.2 (40.6)	>0.05
T_1/2_, h	4.1 (16.2)	4.54 (34.0)	4.83 (30.2)	>0.05
T_max_, h	2.0 (1.0, 3.0)	1.0 (0.625, 1.375)	2.25 (0.5, 4.0)	>0.05
**After green tea**				
C_max_, μg/L	4.30 (58.5)	3.13 (53.4)	4.03 (21.5)	>0.05
AUC_0–24h_, h⋅μg/L	19.06 (47.7)	13.39 (35.6)*	18.29 (42.7)	>0.05
AUC_0–∞_, h⋅μg/L	20.37 (44.9)	14.36 (36.6)	18.98 (42.4)	>0.05
T_1/2_, h	6.15 (19.4)**	4.72 (42.7)	3.98 (31.5)	>0.05
T_max_, h	1.0 (1.0, 2.25)	1.25 (1.0, 2.0)	1.75 (1.5, 3.5)	>0.05
**After soy isoflavones**				
C_max_, μg/L	4.64 (39.1)	3.42 (29.5)	2.86 (20.6)	>0.05
AUC_0–24h_, h⋅μg/L	15.29 (36.7)*	12.63 (31.7)*	17.07 (37.9)	>0.05
AUC_0–∞_, h⋅μg/L	15.61 (36.5)*	13.23 (33.3)*	19.30 (44.0)	>0.05
T_1/2_, h	4.71 (15.5)	4.71 (39.3)	3.90 (49.1)	>0.05
T_max_, h	1.5 (1.0, 1.75)	2.0 (1.25, 2.5)	2.0 (1.125, 2.875)	>0.05

*P < 0.05 vs. baseline; **P < 0.01 vs. baseline.

**TABLE 4:** Effects of the SLCO1B1 521 T > C polymorphism on the pharmacokinetics of simvastatin acid at baseline and after intake of the green tea extract and soy isoflavones.

Variable	521TT (*n* = 6)	521TC (*n* = 8)	521CC (*n* = 4)	*P* values
**Baseline**				
C_max_, μg/L	1.68 (46.3)	1.78 (38.0)	5.11 (35.7)	0.019
AUC_0–24h_, h⋅μg/L	12.48 (30.26)	12.34 (28.03)	40.06 (26.8)	0.001
AUC_0–∞_, h⋅μg/L	14.54 (30.7)	12.87 (29.7)	43.42 (26.4)	0.001
T_1/2_, h	3.63 (25.9)	3.54 (34.4)	5.39(30.2)	0.328
T_max_, h	3.0 (1.75, 4.0)	4.0 (4.0, 4.0)	3.5 (3.0, 4.0)	0.502
**After green tea**				
C_max_, μg/L	1.02 (31.8)	1.73 (33.1)	4.68 (21.9)	<0.0005
AUC_0–24h_, h⋅μg/L	8.45 (31.7)*	14.53 (33.1)	35.43 (25.4)	0.001
AUC_0–∞_, h⋅μg/L	10.0 (31.5)*	16.05 (33.9)	37.24 (25.8)	0.002
T_1/2_, h	5.90 (53.9)	4.86 (38.2)	4.82 (16.2)	0.807
T_max_, h	4.0 (2.0, 4.0)	4.0 (3.0, 4.0)	4.0 (2.875, 4.0)	0.682
**After soy isoflavones**				
C_max_, μg/L	0.93 (16.0)	1.76 (26.5)	4.88 (56.0)	<0.0005
AUC_0–24h_, h⋅μg/L	6.44 (17.5)**	11.0 (27.2)	37.56 (41.8)	<0.00005
AUC_0–∞_, h⋅μg/L	6.88 (18.1)*	11.51 (28.3)	40.45 (37.6)	<0.00005
T_1/2_, h	3.85 (13.4)	3.68 (33.0)	4.41 (40.8)	0.801
T_max_, h	4.0 (2.75, 4.0)	3.5 (2.625, 4.0)	3.5 (3.0, 4.0)	0.823

*P < 0.05 vs. baseline; **P < 0.01 vs. baseline.

Interestingly, subjects with the 521TT genotype also had a significantly reduced systemic exposure to simvastatin acid with green tea extract, but this was not observed with simvastatin lactone, possibly due to wide variation in the systemic exposure to simvastatin lactone among individuals (Table 4). In contrast, subjects with the 521TC genotype but not the two homozygous groups had increased systemic exposure to simvastatin lactone with green tea extract intake. Due to the wide variation in the pharmacokinetics of simvastatin lactone and limited sample size, we cannot be certain if there is an interaction between simvastatin and green tea extract observed in SLCO1B1 genotype subgroup subjects or if this is just a chance finding. There were no statistically significant differences in percentage changes in AUC_0–24h_ of simvastatin lactone and simvastatin acid with green tea extract among the SLCO1B1 genotype groups (Figure 2).

### Effect of the Green Tea Extract and Soy Isoflavones on the Plasma Lipid Profiles and Blood Pressure

The consumption of green tea extract for 2 weeks, but not soy isoflavones, was associated with significant reductions in plasma LDL-C (8.1% [95% confidence interval: −2.0, −14.3%], *P* < 0.01) and total cholesterol (4.8 % [0.4 −10.1%], *P* < 0.05) compared to baseline (Table 5). Reduction in LDL-C with green tea was observed in 15 out of 18 subjects and the change in LDL-C was not affected by the baseline levels. Neither green tea extract nor soy isoflavones influenced the plasma high-density lipoprotein cholesterol (HDL-C) or triglyceride levels or blood pressure in this normotensive group of subjects (Table 5).

**TABLE 5:** Effects of green tea and soy product on plasma lipids and blood pressure.

Parameters	Baseline	After green tea	After soy isoflavones	*P*
Total cholesterol (mmol/L)	4.24 ± 0.73	4.02 ± 0.71*	4.17 ± 0.86	0.111
HDL-C (mmol/L)	1.42 ± 0.24	1.40 ± 0.22	1.37 ± 0.17	0.332
Triglycerides (mmol/L)	0.86 ± 0.25	0.92 ± 0.34	0.90 ± 0.32	0.800
LDL-C (mmol/L)	2.42 ± 0.73	2.21 ± 0.70*	2.39 ± 0.81	0.015
Systolic blood pressure, (mmHg)	113.6 ± 2.0	115.6 ± 2.1	112.8 ± 2.3	0.399
Diastolic blood pressure, (mmHg)	76.6 ± 9.7	77.5 ± 7.8	75.9 ± 9.4	0.779
Pulse rate, (beats per minute)	72.2 ± 10.8	71.8 ± 10.3	68.1 ± 9.4	0.413

P values were assessed by repeated measures analyses of variance (ANOVA) for difference among the three groups. *P < 0.05 vs. baseline.

### Adverse Events

No adverse events were reported during the periods of repeated intake of green tea extract or soy isoflavones or the single doses of simvastatin. The subjects recorded in the food diaries that they complied with the dietary restrictions during the study.

## Discussion

Herbal medicines are often taken concomitantly with therapeutic drugs in many conditions, raising the potential for herb–drug interactions (HDIs). The major pathways leading to HDIs involve the inhibition or induction of CYP-mediated metabolism or drug transporters. The present study showed that administration of ECGC 800 mg per day for 14 days had no significant effect on the pharmacokinetics of simvastatin and simvastatin acid in the overall 18 healthy volunteers, although there was a reduction in systemic exposure to simvastatin acid in the group with SLCO1B1 521TT genotype. The most significant finding of the study is that intake of soy isoflavones with 80 mg daily for 14 days significantly reduced the systemic exposure to simvastatin lactone and simvastatin acid in healthy volunteers with the SLCO1B1 521TT genotype.

This finding is similar to a study which showed that baicalin, derived from the medical plant *Radix scutellariae*, reduced plasma concentrations of rosuvastatin in healthy subjects with haplotypes homozygous for the SLCO1B1 521TT genotype but not in those homozygous for the SLCO1B1 521CC genotype, with intermediate effects in the heterozygotes (28). We previously reported that soy isoflavones did not affect the pharmacokinetics of rosuvastatin (25) but the healthy subjects in that study were not selected for SLCO1B1 genotypes so we cannot exclude the possibility that the number of subjects with the SCLO1B1 521TT genotype was too small to show a significant effect in that previous research.

It has been shown that flavonoids could inhibit multiple ABC efflux transporters, including ABCB1, ABCC2 and ABCG2 (14) as well as the hepatic uptake transporter OATP1B1 (23). However, inhibition of OATP1B1 with soy isoflavones should result in an increased plasma concentration of simvastatin. The previous *in vitro* study examined the effects of individual flavonoids and showed that some of them significantly inhibited [^3^H] dehydroepiandrosterone sulfate uptake in a concentration-dependent manner in OATP1B1-expressing cells (23). This may not be relevant to the present study which used a combination of flavonoids in the soy extract, which may not reach the same concentrations *in vivo* as those used *in vitro*, and also used a different substrate for OATP1B1.

It was previously demonstrated that feeding rats diets containing soy protein isolate (SPI) results in alterations in expression and inducibility of a number of CYP enzymes, including CYP1A1, CYP1A2, CYP2B1, and CYP2C11 (16, 29). In addition, it was shown that there was a significant elevation in expression and glucocorticoid-inducibility of hepatic CYP3As after feeding SPI-containing diets to rats and mice and after feeding soy infant formula to neonatal piglets (17, 18). The nuclear hormone receptor, PXR regulates multiple drug metabolizing enzymes and transporters including CYP3A4 and activation of PXR by endogenous and exogenous chemicals results in induction of drug metabolism. CYP3A4 is the most important phase I drug metabolizing enzymes that is responsible for the metabolism of approximately 50% of all prescription drugs including simvastatin. Li et al. demonstrated that the soy-associated isoflavones, genistein, daidzein and equol, activated both mouse and human PXR and subsequently upregulated CYP3A enzymes (18). It is therefore possible that the interaction between soy isoflavones and simvastatin is mediated via activation of PXR.

In addition, other studies have shown that soy isoflavones, genistein or daidzein, can activate PPARα and PPARγ and regulate LXR activity indirectly by promoting the phosphorylation of LXRα and LXRβ, leading to differential expression of genes regulated by LXR (20, 30). These nuclear receptors can modulate CYP3A4 and multiple influx and efflux drug transporters suggesting that there may be complex interplays between nuclear receptors, CYP3A4 and drug transporters responsible for the observed interaction between soy isoflavones and simvastatin.

Considering that soy isoflavones significantly reduced the systemic exposure to simvastatin acid, but not the lactone, this may suggest the effect is through a transporter rather than the CYP enzymes as the lactone forms of most statins are metabolized more extensively than the active acid forms (31). Furthermore, the effect was only significant in the SLCO1B1 521TT genotype group with the most active form of the OATP1B1 transporter, suggesting the effect may be mediated by increasing expression or activity of this transporter. There was also a significant reduction in the AUC for simvastatin lactone with soy isoflavones and a significant reduction in the AUC for simvastatin acid with green tea extract in this genotype group. The effect could be mediated via activation of PXR to increase expression of OATP1B1 or a direct effect on the transporter activity by certain flavonoids.

As mentioned above, simvastatin acid is also a substrate for the efflux transporters, ABCG2, ABCB1 and ABCC2 (7, 12), and less active forms of ABCG2 are associated with increased systemic exposure to simvastatin acid (8). It is therefore possible that activation of ABCG2, ABCB1 or ABCC2 would reduce the AUC for simvastatin acid but it seems unlikely that the effect would be influenced by the SLCO1B1 genotype. Likewise, other OATP transporters expressed in hepatocytes and enterocytes such as OATP1B3, OATP2B1 and OATP1A2 may play a minor role in the disposition of simvastatin acid (9), but effects on these are unlikely to be SLCO1B1 genotype-dependent.

A previous case report documented that consumption of green tea might be associated with increased systemic exposure to simvastatin lactone and acid and appeared to trigger statin muscle intolerance in a 61-year-old man with hypercholesterolemia who developed muscle symptoms while receiving low doses of various statins (13). In addition, an *in vitro* study demonstrated that green tea extract (containing EGCG, EGC, EC, and ECG at 43.3, 24.8, 9.7 and 1.7%, respectively, with a total catechin content of 86.5% w/w) weakly to moderately inhibited CYP3A activity in a non-competitive manner as evaluated by midazolam 1′-hydroxylation in rat hepatic microsomes (32). A single oral dose of green tea extract (400 mg/kg) 30 min before simvastatin administration was associated with significantly increased AUC_0–6h_ of simvastatin by 3.4-fold but had no effect on t_1/2_ in rats suggesting that green tea extract did not affect the elimination of simvastatin (32). The AUC_0–6h_ of simvastatin acid was increased by 2.0-fold in this animal study but this was not statistically significant due to large inter-individual variations (32).

The discrepancies between our study and the previous *in vitro* and animal studies may be due to various factors. Firstly, the *in vitro* and animal studies generally use very high doses or concentrations of green tea extract which may not be clinically relevant and may have different effects on drug metabolizing enzymes and transporters. It has been estimated that a freshly brewed cup of green tea may contain 130–180 mg of EGCG (33). The present study used an 800 mg dose of green tea extract, which is equivalent to about 5–6 cups of green tea and may be more relevant to the normal intake, although giving the extract as a single dose will result in higher maximum concentration of catechins than normal consumption of green tea as a beverage throughout the day. Secondly, the previous experiments with green tea extract were performed with a single dose of green tea extract or a single cup of green tea and these may have different effects from the multiple doses used in the present study. This study used an extract which contained predominantly EGCG, which is the most abundant catechin in green tea (up to 80%), whereas other studies may have used other green tea extracts or green tea drinks contain a different mixture of catechins which may again also have different effects on drug metabolizing enzymes and transporters.

In a previous study we showed that the same extract of green tea reduced the systemic exposure to rosuvastatin by about 20% when the extract was given daily for 2 weeks and simultaneously with the dose of rosuvastatin (25). That effect may have been due to activation of liver uptake by OATP1B1 or OATP1B3, or inhibition of intestinal uptake by OATP2B1 or OATP1A2. It has been shown that EGCG can inhibit OATP1A2- and OATP2B1-mediated uptake of estrone-3-sulfate in a concentration-dependent manner in cells expressing these transporters (34). Green tea taken as a drink reduced the systemic exposure to nadolol by 85% in healthy volunteers, which was thought to be due to inhibition of OATP1A2-mediated intestinal uptake of nadolol which was supported *in vitro* studies (35).

Moreover, EGCG was shown to activate OATP1B3 in *in vitro* studies (36). Transporters such as ABCB1 and possibly hepatic OATP1B transporters can be induced by various compounds by activating PXR (37). The effect of the green tea extract used in the present study to reduce the systemic exposure to rosuvastatin in our previous study and possibly reduce the systemic exposure to simvastatin in the SLCO1B1 521TT genotype group in the present study may be due to activation of liver uptake transporters and such effects are known to vary with different substrates (37). Increased liver uptake of these statins may result in increased LDL-C lowering effects and it would be interesting to examine this.

## Limitations

There are some limitations in this study. It is well known that HDIs may depend on the dosage of herbs used and, in this study, we only investigated one dosage for each herbal product. The dosage was chosen to correspond with a high intake of the natural substances in food or beverages. Secondly, there may be a physical reaction between the herbal extracts and the drug because the subjects took the herbal extracts and simvastatin simultaneously on the dosing day to try to identify the maximum interaction between the herbs and drug. It was shown that the bioavailability of sunitinib in rats was reduced when taken together with EGCG but not when the EGCG was taken 8 or 4 h before sunitinib, which appeared to be due to a physical reaction between the two compounds when taken together (38). Thirdly, although we instructed the subjects to follow the dietary restrictions, this relied on the subjects’ cooperation and honesty and may not be entirely reliable, but it was a practical way to conduct the study.

## Conclusion

Repeated green tea catechin administration at a daily dose of about 800 mg EGCG for 2 weeks had no significant overall effect on the pharmacokinetics of simvastatin in healthy volunteers but appeared to reduce the systemic exposure to simvastatin acid in subjects with the SLCO1B1 521TT genotype. Soy isoflavones at a dose of approximately 80 mg daily for 14 days was associated with reduced systemic exposure to simvastatin and simvastatin acid and this interaction appeared to occur in subjects with the SLCO1B1 521TT genotype but not in subjects with the 521C variant allele. Further studies are needed to investigate the underlying mechanisms responsible for these observed interactions and to assess the clinical relevance of these interactions in patients receiving long-term simvastatin.

## Data Availability Statement

The original contributions presented in the study are included in the article/supplementary material, further inquiries can be directed to the corresponding author/s.

## Ethics Statement

The studies involving human participants were reviewed and approved by the Joint Chinese University of Hong Kong-New Territories East Cluster Clinical Research Ethics Committee. The patients/participants provided their written informed consent to participate in this study. Written informed consent was obtained from the individual(s) for the publication of any potentially identifiable images or data included in this article.

## Author Contributions

WZ and MH analyzed the data and wrote the manuscript. BT designed the research project. MH, BT, HL, EW, CL, CW, and CH performed the experiments. BT and CH revised the manuscript. All authors contributed to the article and approved the submitted version.

## Conflict of Interest

The authors declare that the research was conducted in the absence of any commercial or financial relationships that could be construed as a potential conflict of interest.

## Publisher’s Note

All claims expressed in this article are solely those of the authors and do not necessarily represent those of their affiliated organizations, or those of the publisher, the editors and the reviewers. Any product that may be evaluated in this article, or claim that may be made by its manufacturer, is not guaranteed or endorsed by the publisher.
